# Understanding loneliness: a systematic review of the impact of social prescribing initiatives on loneliness

**DOI:** 10.1177/1757913920967040

**Published:** 2021-06-23

**Authors:** GY Reinhardt, D Vidovic, C Hammerton

**Affiliations:** Department of Government, University of Essex, Wivenhoe Park, Colchester CO4 3SQ, Essex, UK; University of Essex, Colchester, UK; University of Essex, Colchester, UK

**Keywords:** public health, wellbeing, loneliness, evaluation, social prescribing, social model of health

## Abstract

**Aims:**

The aim of this systematic literature review is to assess the impact of social prescribing (SP) programmes on loneliness among participants and the population.

**Methods:**

We followed Preferred Reporting Items for Systematic Reviews and Meta-Analyses (PRISMA) guidelines to search EBSCOHost (CINAHL Complete, eBook Collection, E-Journals, MEDLINE with Full Text, Open Dissertations, PsycARTICLES, and PsycINFO), UK National Institute for Health and Care Excellence (NICE), Web of Science Core Collection, and grey literature. We included studies measuring the effectiveness and impact of SP programmes in terms of loneliness. We excluded systematic reviews and studies without evaluations. Due to the absence of confidence intervals and the low number of studies, we conduct no meta-analysis.

**Results:**

From 4415 unique citations, nine articles met the inclusion criteria. The studies do not use uniform measures or randomised samples. All nine studies report positive individual impacts; three report reductions in general practitioner (GP), A&E, social worker, or inpatient/outpatient services; and one shows that belonging to a group reduces loneliness and healthcare usage.

**Conclusion:**

The findings of this systematic review indicate that individuals and service providers view SP as a helpful tool to address loneliness. However, evidence variability and the small number of studies make it difficult to draw a conclusion on the extent of the impact and the pathways to achieving positive change. More research is needed into the impact of SP programmes on participants, populations, and communities in terms of loneliness, isolation, and connectedness, especially in light of the surge in SP activity as a key part of pandemic response.

## Introduction

Addressing loneliness has been part of the public health agenda in countries like the UK and Canada since before the COVID-19 (coronavirus disease 2019) pandemic. Linked to numerous physical and mental health conditions, adverse effects of loneliness have been observed in educational, workplace, and wider community settings. Loneliness is also linked to increases in health and social care usage^1^ due to increased mortality, blood pressure, depression and anxiety, and decreased mobility and quality of life.^2,3^

*Loneliness* is a subjective, unwelcome feeling of lack or loss of companionship that occurs when there is a mismatch between the quantity and quality of social relationships that a person has, and those that person wants.^4,5^ Though often associated with isolation, loneliness is distinct in that it is a feeling, while *isolation* is an objective measure of the number and quality of contacts that one has.^6^ Thus, it is possible to be lonely while surrounded by others, or to have very few social contacts but not feel lonely. Loneliness can also perpetuate itself, disrupting social interaction and integration and reducing one’s healthy relationships.

The need to address loneliness has become all the more urgent since the onset of COVID-19, as individuals and organisations have sought to maintain social connection amid restrictions on physical interaction. Social care and public health agencies have distributed digital tablets, created online forums, and hosted virtual events in attempts to help keep people connected. To help inform efforts to address this need, we present this systematic review of evaluations of interventions designed to tackle loneliness.

Specifically, we focus on interventions known as *social prescribing* (SP). Concurrent with increased awareness about loneliness and its threat to public health, practitioners, policy makers, and researchers around the world have been calling for a fundamental change in healthcare systems to implement person-centred, holistic care. This social model of health has been adopted in various forms in Canada,^7^ the UK,^8^ and the US,^9^ and SP programmes are a part of it.

The example of the UK can help illustrate the believed linkages between loneliness and SP. In 2018, the UK Government published the Loneliness Strategy. Since then it has devoted significant resources to combatting loneliness and improving individual and community wellbeing, including engaging with numerous charities, to demonstrate its commitment to tackling loneliness and promoting social connections.^10^ In 2019, the UK Government launched Universal Personalised Care (UPC), a system designed around six key pillars meant to give individuals choice and control over their mental and physical health. UPC was intended to help the UK health system enhance value for money and improve overall health and wellbeing, including through the reduction of loneliness.^11^

The fourth UPC pillar is centred on SP. SP programmes employ link workers (also called community connectors, community navigators, and/or village agents) to guide participants to co-develop personalised solutions for their own health. As an asset-based, collaborative approach, SP programmes are designed to identify needs and resources, promote and develop individual and community capacities, and ameliorate symptoms and consequences of poor health.^12^ With the UPC launch, the UK Government committed to reaching more than 900,000 people through SP by 2023–2024. Through this commitment, it was intended to also reduce loneliness and improve public health.^13^

In the UK, there are four sectors associated with SP interventions. First, some general practitioner (GP) practices within the health sector are actively engaging link workers to accept referrals and work individually with people and families. Second, organisations in the voluntary and community service (VCS) sector individually with people and families supply an array of innovative and engaging activities for them to access for support and connection. This sector employs link workers directly and supplies many of the services that other link workers recommend.

Third, social care services offer complementary support to vulnerable and elderly people and families by developing the market for SP, by commissioning and funding community activities, and by supplying SP through local authorities and/or councils. And finally, Departments of Public Health provide SP services as they seek to enhance the health of the population as a whole, providing evidence on the position and quality of public health and filling gaps in the availability of services. One person might therefore encounter SP through any one of these sectors, or through an integrated care system that combines these sectors to offer a holistic approach to care and wellbeing.

The variety of ways in which SP can be offered means there can also be a variety of aims and goals between programmes. Many SP services run out of GPs, for example, are interested in how SP can improve health and reduce the burden on the healthcare system; these programmes are overseen by the National Health Service (NHS) in the UK. Those SP services run by local councils might be overseen by Departments of Public Health, Social Services, or Public Safety; their key goals could be improved public health or security. SP programmes implemented by the VCS tend to be focused on individual wellbeing.

The diversity of goals of SP programmes, combined with the recent surge in SP in the UK and person-centred care around the world, raises questions regarding the effectiveness and impact of these models on mental and physical wellbeing in general, and on loneliness in particular. As a collaborative effort between public, private, and third sector organisations, SP is well-suited to provide person-centred healthcare and improve public health outcomes. Yet, we need more information about SP outcomes if we are to understand the extent to which they affect loneliness.^11,14,15^ This systematic review therefore focuses on interventions designed to reduce loneliness, detailing methods used to differentiate and define individuals’ health conditions and needs, as well as the impact of the SP interventions employed to reach lonely individuals.

We analyse research into SP schemes in the UK and internationally over two decades. In contrast to previous reviews,^16,17^ we follow 2019 NHS England and Drinkwater et al.’s recommendations^8,13^ to evaluate the outcomes of SP-type programmes by assessing the impact of a programme at three levels: the person, the health and social care systems, and the community. These three levels of measurement capture a range of potential impacts and help us understand the effects of SP as an approach to engage and empower individuals and communities to co-design health plans, reduce loneliness, and promote public health.

As we detail below, our work yields evidence on the use of SP initiatives to address loneliness in the UK, but does not end up including evaluations of initiatives from other countries, despite the fact that we did not restrict our search geographically. We offer two potential explanations for this outcome. First, the use of SP to address loneliness is still a novel concept; SP programmes are often evaluated in terms of other aims and the UK is the only context that measures outcomes in terms of loneliness.

Second, we focus on the term ‘social prescribing’ for our search to isolate an increase in the literature on SP across the globe (see Box 1). As a result, our findings do not include research on other similar programmes, such as Local Area Coordination, Community Navigation, or Village Agents, unless they also include the ‘social prescribing’ moniker. To the extent that this alterative terminology is more commonly used in other contexts, these programmes highlight parts of the world or health systems excluded from our search.

**Box 1:** Search strategy used in the systematic review of social prescribing programmes on loneliness

(social prescri* AND lonel*) AND (interven* OR evaluat* OR program*) (social prescri* AND connect*) AND (interven* OR evaluat* OR program*) (social prescri* AND well-being) AND (interven* OR evaluat* OR program*) (social prescri* AND wellbeing) AND (interven* OR evaluat* OR program*) (social prescri* AND well being) AND (interven* OR evaluat* OR program*) (social prescri* AND isolat*) AND (interven* OR evaluat* OR program*)

## Methods

We followed the Preferred Reporting Items for Systematic Reviews and Meta-Analyses (PRISMA) guidelines and Petticrew and Roberts’ advice in conducting our review.^18,19^ Our protocol has not been registered on the PROSPERO register of systematic reviews, but is available from the authors upon request.

## Design and Sample

### Research strategy

We conducted a comprehensive search in social science and public health repositories to identify existing studies on the effect of SP on loneliness. Through EBSCOHost, we searched nine bibliographic databases (CINAHL Complete, eBook Collection, E-Journals, MEDLINE with Full Text, Open Dissertations, PsycARTICLES, and PsycINFO), as well as the UK National Institute for Health and Care Excellence (NICE) and Web of Science Core Collection, for research published in the English language from 1 January 2000 to 30 November 2019. EBSCOHost and Web of Science Core Collection include many peer-reviewed, high-quality scholarly journals published worldwide (including open access journals) as well as conference proceedings and books. NICE provides access to numerous social science and medical journals such as *The BMJ*, as well as links to work published by think tanks, non-profit organisations, community health groups, and the government.^20^ We searched for combinations of SP, evaluation, and potential impact (Box 1).

As mentioned above, the UK commonly uses the term ‘social prescribing’ to characterise an asset-based model of service delivery. Models such as Local Area Co-ordinators, community navigators, or village agents are also based on the social model of health to connect people to their communities and universal services, often through voluntary sector services. We chose to focus on the term ‘social prescribing’ to recognise and investigate the rise of literature and programming across the globe using this term.

### Inclusion criteria and data collection

Two researchers screened the identified abstracts. Studies were eligible for inclusion if they included a programme or initiative designed to offer person-centred care. We included both peer-reviewed and grey literature reporting studies evaluating the impact of one or more interventions on one or more levels of analysis: the person, the health and care system, or the community. When doubt or disagreement occurred on whether an article met the inclusion criteria, the article was moved to the next stage of screening. After initial screening, we appraised the studies to determine whether the programmes were designed to address loneliness either as a sole characteristic or as one of several. We excluded systematic reviews, studies that did not include an evaluation of an intervention, and instructional materials that gave advice on how to conduct SP programmes.

### Data synthesis

The researchers independently assessed the full text of potentially eligible studies and extracted details of the studies into a database. The data collected were as follows: country and area of the programme or intervention; aim of the programme; type of programme (signposting, light, medium, or holistic);^21^ whether programme was implemented through GPs, the voluntary sector, social care workers, or an integrated care system; study time frame and data collection period; study type and sampling method; description of study population (age, gender, location, health characteristics); sample size; analytical method; evaluation design (randomised, control group present, pre/post testing); and outcome/impact reported on the person, the health and social care system, and/or the community. The outcome of interest for the review was loneliness.

## Results

### Study identification

**Figure 1:**
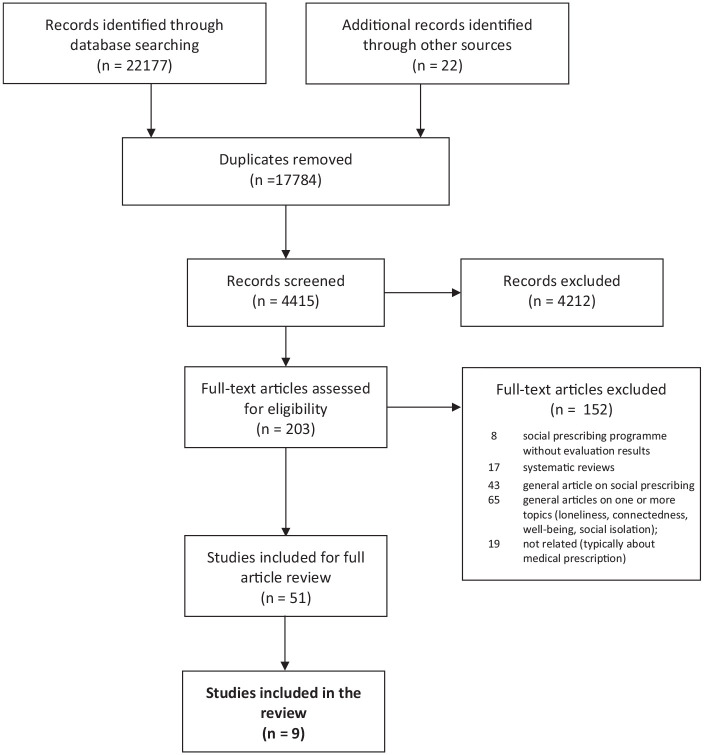
PRISMA flow diagram of the systematic review of social prescribing programmes designed to address loneliness across the globe

Our search yielded 22,199 references, of which 4415 were unique entries. Figure 1 illustrates our process. We excluded 4212 articles after screening titles and abstracts. Of the 203 references that potentially met the inclusion criteria, 152 were excluded for different reasons (Figure 1). Left with 51 studies, we excluded 42 because they were not designed to address loneliness. This process left nine articles for review. Of these, three were designed to address loneliness as a sole characteristic and six were designed to address loneliness in addition to social isolation, wellbeing, and/or connectedness. Study results are highly heterogeneous due to variability in sampling methods and the definition of loneliness. In view of this heterogeneity and the absence of confidence intervals, we do not attempt meta-analysis.

### Study characteristics

Two publications are peer-reviewed articles and seven are study reports. The nine articles are based on nine SP initiatives conducted in the UK from 2014 to 2019 (Table 1). Eight of the studies include a total of 12,359 study participants, plus at least 9000 in the ninth study that does not report exact numbers. Three of the studies include individuals aged 16 years or older,^22,23^ one has participants aged 29–85 years,^15^ one has participants aged 36–40 years,^24^ one has participants aged either below 30 or above 60 years, and one has participants aged above 65 years.^25^ Two of the studies do not specify participants’ ages.^26,27^

**Table 1:** Systematic review of social prescribing (SP) programmes designed to implement loneliness across the globe, during 2000–2019 period

SP programme name Location	Aim of SP initiative Sample characteristics SP programme participant characteristics	Date of SP programme Evaluation research design, method Measures of loneliness	Impact
**Programme name:** Dudley Community and Voluntary Services^23^ **Location:** Dudley, UK	**Aim:** Connecting people, helping them find purpose in their lives. Reducing patient demand on GP and A&E. **N** = 2720 **Age:** 16+; 60% aged 64+, 37% aged 24–63, with remaining 3% between 16 and 23. **Participants:** Patients who frequently attend their GP practice, are in top 2% at high risk of admission, and any vulnerable person in need of non-clinical support as identified by their GP. Isolation was the highest reason for referral.	**Date:** September 2014 to August 2018 **Design and method:** Pre/post and case studies; surveys. **Measurement:** Isolation/Loneliness used interchangeably. Six indicators of social contact, no justification.	**Person:** Number of people feeling lonely and without enough contact reduced by 46% (87–46). Number of people not feeling lonely and with enough contact increased by 39% (97–135). **System:** *GP visits*: Of the 43 GP practices, 6-month post-programme 8 practices had an increase of 63 additional consultations in total, 34 had a decrease of 2125 in total, and 1 had no change. Most healthcare providers reported the key benefit of SP to be reduction in participants’ isolation and loneliness. *A&E*: 14% reduction in participants’ attendance after 6 months, 17% reduction after 12 months. *Inpatient admissions*: 14% reduction after 6 months, 15% reduction after 12 months. **Community:** Not assessed.
**Programme name:** Connecting Communities Programme^22^ **Location:** 30 locations across the UK	**Aim:** To re-connect lonely or socially isolated people to their communities and provide emotional and practical support. To offer person-centred support to build self-confidence and resilience and help people forge social connections. **N** = Over 9000 (no exact number). **Age:** 51% aged below 70; 82% of the sample was classed as being lonely (UCLA scale) at the start of the programme. **Participants:** Statutory health and care services such as the NHS (22%) and local authorities (19%), and others such as family and friends, private organisations, and self-referral.	**Date:** May 2017 to December 2018 **Design and method:** Pre/post; surveys. **Measurement:** 3-item UCLA Loneliness Scale.	**Person:** 69% less lonely, 27% no change, 4% more lonely. Participants below 60 years had more improvement in loneliness compared to those above 60 years. Greater impact on participants identified as being in a life transition (health issues, mobility limitations, new child, recent bereavement, divorce/separation, retirement, children moving out) than on those not experiencing transition. **System:** Not assessed. **Community:** Not assessed.
**Programme name:** Social Cure^15^ **Location:** East Midlands, UK	**Aim:** To determine which social factors are central to understanding SP, how SP is experienced across participants and those who deliver the programme, provide evidence base for impact of SP and the consequences for patient’s healthcare use. **N** = **Study 1:** 19 participants; 7 GPs referring participants; 3 health coaches; and 6 link workers working with participants. **N** = **Study 2:** 630 participants at a 4-month follow-up after initial referral assessment. **Age:** 29–85 (average age: 60.4). **Participants:** Referred by GP or self-referral. 37% (*n* = 7) multiple/complex needs including loneliness. 53% (*n* = 10) weight loss + multiple needs including loneliness. Social cure received 1483 referrals and supported approximately 650 patients.	**Date:** November 2017 to February 2019 **Design and method:** Pre/post; Study 1: semi-structured interviews; Study 2: longitudinal survey. Considers participants’ gender, age, relationship status, employment status, education levels, and pre/post-programme levels of loneliness, community belonging, and healthcare usage to test the pathway between the programme designed to address loneliness and healthcare usage outcomes. **Measurement:** 8-item UCLA Loneliness Scale (ULS-8).	**Person:** Loneliness and social isolation are key threats to public health and can be addressed through SP. Interviews revealed that being a part of a group (family, community, and volunteering group) and feeling that one belongs to a community helps people feel less lonely. Participants report that having a positive relationship with link workers has helped them build self-confidence, which in turn has helped them address their experiences of loneliness. Group membership alone is not directly and significantly related to primary case usage. Sense of community belonging should be considered when examining this pathway. **System:** *GP visits*: GPs, health coaches, and link workers recognise the limitations of the ‘traditional medical model’ and express concerns over addressing loneliness with medical provisions. GPs view SP as best model to address loneliness and reduce its negative health impacts. **Community:** Primarily focuses on understanding how community resources can be used to reduce loneliness and healthcare usage, and less so on impact of the programme on community.
**Programme name:** Museum on Prescription^29^ **Location:** London and Kent, UK	**Aim:** To support the wellbeing of socially isolated and lonely older people by assessing the impact of participation in 12 Museum on Prescription programmes. **N** = 20 **Age:** 65–94 **Participants:** Selected from a pool of 155 individuals who self-identified as lonely or socially isolated.	**Date**: Not specified. **Design and method:** Case study; interviews, theory building using grounded theory analysis and inductive approach. **Measurement:** R-UCLA Loneliness Scale.^31^	**Person:** Participants report feeling less lonely, more able to develop meaningful connections and friendships, greater confidence, more mental stimulation, and more feelings of happiness. **System:** Not directly assessed. Theoretical discussion supports prevention-based initiatives. Offers framework for considering individual characteristics and life experiences when developing community-based later-life social interventions. **Community:** Not directly assessed. Theoretical discussion suggests that opportunities to develop new connections, engage in new experiences, and become more socially engaged could inspire participants to make a positive change in their own communities.
**Programme name:** Not reported^26^ **Location:** Unnamed local authority area, UK	**Aim:** Pilot was developed with an aim to discover sustainable and strategic approach to commissioning services that supported primary care objectives. The aim of the evaluation was to examine the changes in the healthcare use and changes in participants’ wellbeing. **N1** = 108 (consists of 42 opted to participate in a ‘pump-priming’ component; 62 opted out of ‘pump-priming’ portion) **N2** = 280 participants from pilot only assessed for their wellbeing. **Age:** not specified. **Participants:** Referred by GP.	**Date:** Not specified. **Design and method:** Pre/post; surveys and interviews with 44 carers, commissioners, and providers **Measurement:** Not provided.	**Person:** Quotations evidence a reduction in loneliness and social isolation. **System:** *A&E*: 20% reduction in number of visits in 12-month post-participation period. Participants in pump-primed service experienced greater reduction in this service demand compared to those who opted out – an average difference of 0.22 attendances per participant. *Inpatient admissions*: 21% reduction in the number of admissions in 12-month post-participation period. Participants in pump-primed service experienced greater reduction in inpatient service demand compared to those who opted out – an average difference of 0.10 attendances per participant. *Outpatient appointments*: 21% reduction in the number of admissions in 12-month post-participation period. Participants in pump-primed service experienced greater reduction in outpatient service demand compared to those who opted out – an average difference of 0.31 attendances per participant. **Community:** Reports that unspecified number of participants became volunteers engaged in wider voluntary and community activity once pilot concluded.
**Programme name:** Doncaster Social Prescribing^25^ **Location:** Doncaster, UK	**Aim:** To help with the effects of long-term physical and mental health conditions. **N** = 1054 **Age:** More than half of the sample aged 60 and above, around one-quarter aged above 80, and the rest were ⩽30. **Participants:** Referred by GP, community nurses, and pharmacists.	**Date:** August 2015 to June 2016 **Design and method:** 254 participants completed an intake questionnaire and either 3- or 6-month follow-up (*n* = 215). Pre- and post-programme comparisons. **Measurement:** Adult Social Care and Public Health Outcome Framework (ASCOF/PHOF) is used to assess the levels of social isolation and loneliness (used interchangeably).	**Person:** Participants felt less isolated or alone post-participation, ‘feeling like they had someone they could turn to’. No direct evidence or discussion on the loneliness measure that was administered. 19% increase in people having ‘enough social contact’. **System:** *GP visits*: 68% report reduction in GP appointments; 15% report increase; 17% no change. *A&E*: 7% report reduction in attendance; 1% report increase; 92% no change. *Inpatient admissions*: 9% report reduction in stays; 3% increase; 90% no change. *Social care*: 3% report reduction in contacts with social worker; 97% report no change (3% of sample reported having a contact with social services 3 months prior to start of the programme). **Community:** A non-specified number of volunteers have found employment since being involved the project. 88% report greater awareness of the services and support available.
**Programme name:** Age UK’s Cascade Training^27^ **Location:** Across England, UK	**Aim:** To evaluate the effectiveness of the consultancy support, training, and training packs. To engage older people in activities to improve health and wellbeing, reduce the demand for health and social care, and help delivery organisations to train volunteers to engage hard-to-reach, older people. **N** = 5368 older people; 1382 volunteers; 75 delivery organisations **Age:** Not reported. **Participants:** Not reported.	**Date:** 2013 to 2015 **Design and method:** Interviews, surveys, focus groups, documentary analysis, follow-up with organisations’ data collection teams. **Measurement:** Not reported.	**Person:** Service delivery staff report positive impact of SP on loneliness, recommended that training manuals include measures to address loneliness and social isolation. 95% of staff report ability to support more older people as a direct result of the programme. 58% of volunteers report positive impact on their own mental health and wellbeing. **System:** Positive impact on care home services, improving residents’ quality of life. **Community:** Delivery organisations report expanding services and creating new activities due to programme. Programme brought together housing associations, sheltered housing and care home staff, healthcare providers, faith-based organisations, and local charities, which has a positive impact on community engagement. Participants report interest in helping others and sharing information, thereby expanding community capacity to respond to challenges.
**Programme name:** Social Prescribing Pilot^28^ **Location:** Rotherham, UK	**Aim:** To assist GPs to meet the non-clinical needs of patients with complex long-term conditions. **N** = 559; *n* = 451 (6 months post-referral) *n* = 108 (12 months post-referral). **Age:** 87% aged 60–69; 75% aged 70–79; 47% aged 80–89; 10% aged ⩾90. **Participants:** GP-led Integrated Case Management Teams referring patients through GPs to Community and Voluntary Services	**Date:** September 2012 to April 2014 **Design and method:** Case studies; interviews with participants (17) and with individuals delivering service (10). **Measurement:** none.	**Person:** Participants report feeling like they belong more to a community and that they have enjoyed more social contact, with researchers drawing conclusions on reduction in loneliness and isolation. **System:** *GP visits*: not reported. *A&E*: 38% of participants report a reduction in attendance 12 months post-referral, 25% report reduction 6 months post-referral. *Inpatient admissions*: 40% reduction 12 months post-referral, 24% 6 months post-referral. *Outpatient admissions*: 47% reduction 12 months post-referral, 30% 6 months post-referral. Impact greater for participants referred to other funded services (48% reduction in inpatient admissions, 43% in A&E visits, 12 months post-referral). **Community:** Small organisations without previous access to NHS funding were able to access it for the first time, which enhanced their provision and improved their sustainability.
**Programme name:** Wellspring Wellbeing Programme^24^ **Location:** Bristol, UK	**Aim:** To connect, be active, take notice, keep learning, and give. **N** = 128 **Age:** 36–40 **Participants:** Referred by GP.	**Date:** May 2012 to April 2013 **Design and method:** Pre/post; interviews, and questionnaires. **Measurement:** Hawthorne Friendship Scale and Wellspring Wellbeing Questionnaire to assess loneliness and social isolation.	**Person:** Number of socially isolated (lonely) Friendship Scale measure decreased from 67.8% (*n* = 59) to 33.4% (*n* = 15) 3 months post-programme. **System:** *GP visits*: 60% of participants reduced GP attendance rates 12 months post-intervention, 26% no change, 14% increase. **Community:** Not assessed.

NHS: National Health Service; GP: general practitioner; UCLA: University of California, Los Angeles; R-UCLA: Revised UCLA Loneliness Scale; ASCOF/PHOF: Adult Social Care and Public Health Outcome Framework.

Six studies employ a pre/post design^15,22–26^ and three report case studies with evidence taken at one point in time.^27^–^29^ None of the studies consider a control group. Three studies conduct surveys only,^22,23,25^ two conduct interviews only,^28,29^ and four mix the two methods.^15,24,26,27^ Five studies are conducted with SP recipients only,^22^–^25,29^ while four also present information gathered from link workers, volunteers, and GPs who deliver the programme.^15,26^–^28^

Four studies either do not distinguish between *loneliness, connectedness*, and *isolation* or use the terms interchangeably.^23^–^25,28^ Five studies define and justify how they measure loneliness.^30^ Of these, two use the 8-item UCLA (University of California, Los Angeles) scale,^5,15,29^ one uses the 3-item UCLA scale,^22,31^ one uses the Adult Social Care and Public Health Outcome Framework,^25,32^ and one uses the Hawthorne Friendship Scale.^24,33^ Four either do not report how they assess loneliness^26^–^28^ or do not report how their assessments were designed or chosen.^23^

### Impact on the individual

All nine studies report positive impact on the individual social prescribing participant. Impact areas in addition to loneliness include healthcare service usage^15,23^–^29^ and social care service usage.^34^ Two studies report individuals expressing in interviews that they feel less lonely/more connected to others^28,29^ and two report changes in loneliness scores across the participant sample.^22,23^ The highest impact reported is 69% of individuals feeling less lonely (UCLA 3-question version).^22^

Two of the studies examine the extent to which age might impact social prescribing programme implementation and loneliness.^15,22^ One of these studies reports greater improvements in loneliness for individuals below 60 years of age in comparison with those aged 60 and above.^22^ One examines age as a contextual factor determining the pathway between a social prescribing programme and healthcare usage outcomes.^15^

### Impact on the health and care system(s) and community

Evaluation of the impact on health and care services is primarily focused on documenting numbers of GP visits, Accident and Emergency (A&E) visits, inpatient admissions, and outpatient admissions. Three studies report GP visit reduction ranging from 20% to 68%.^23^–^25^ Two studies report an increase in GP and A&E visits following programme implementation.^23,25^ One study reports a 3% reduction in the number of contacts participants had with a social worker following programme implementation.^25^

One study links measures of community belonging to system and individual health measures. It shows that being a member of a group (family, community, and volunteering group) positively predicts one’s sense of community belonging, which in turn predicts reduced loneliness and reduced healthcare usage.^15^ This study also reports that GPs view social prescribing as the best model to address loneliness and its negative impact on health.^15^

The nine studies diverge in how they assess impact on the community. One study reports greater participant awareness of available services and support.^25^ Two report organisations expanding their service capacity.^27,28^ One reports a greater sense of community connectedness.^15^ Five studies do not address programme impact on the community.

## Discussion

Nine studies in this systematic review gauge the effects of social prescribing on loneliness. Overall, social prescribing models designed to address loneliness have been largely viewed as helpful by both participants and service providers. Participants report feeling less lonely and more connected to others. Participants feel good about their relationship with a link worker and appreciate the service delivery model. GPs, volunteers, and delivery service members view social prescribing as a valid model to deliver comprehensive, people-centred, and integrated care, and some GPs view social prescribing as the best possible approach to successfully address loneliness. The positive impact appears as a large percentage of reductions in GP, A&E, and inpatient and outpatient services following programme implementation. However, the variability and paucity of evidence and lack of control group comparisons make it difficult to draw conclusions regarding the impact of the social prescribing model on loneliness in particular, or on public health in general.

### Quality of impact evidence

Largely insufficient supporting evidence makes it difficult to quantify the impact of these programmes and interventions. The nine studies primarily rely on a pre/post-study design, lack control group comparisons, and neglect to consider the potential influence of other conditions on the outcomes of interest. Study participants are typically selected through GP referrals, a selection that is not systematic or explained. In addition, several studies do not provide a clear definition or a measure of loneliness and often use social isolation and loneliness interchangeably.

Despite programme participants reporting various health and social care needs, only one study examines social care outcomes.^25^ Because these initiatives are designed to address loneliness, the lack of attention to social care usage should be troubling. Without knowing the extent to which social service usage is affected, it is impossible to know whether social prescribing is meeting individual needs, changing referral rates, or yielding cost savings. We therefore have little to learn from these studies regarding the relationship between loneliness and social care usage, and even less regarding how the social prescribing person-centred approach might affect that relationship.

Because social prescribing programmes are meant to deliver person-centred care, it is natural to be concerned with the impact of such programmes on individuals. Since person-centred care is intended to account for social relationships and overall community connectedness, however, the impact of social prescribing on communities should also be considered. It is therefore surprising how few of the existing studies examine the relationship between social prescribing programmes and the communities in which they operate.

The NHS England has proposed a more systematic approach to capture community impact, which they assert should be done by assessing the capacity of community groups to manage social prescribing referrals.^8,13^ Given that community connectedness has also been linked to economic productivity, crime rates, civic behaviour, and empowerment, these are also community attributes wherein social prescribing programme impact could be measured.^35^

### Implications for research and/or practice

A significant contribution of the social prescribing approach to person-centred care is that it allows services users and providers to co-design a model of care tailored to individual needs. The relationship participants and social prescribers develop over time is a potentially useful way for individuals to become less lonely, reconnect with their community, and improve their physical and mental wellbeing. The social prescribing model has the capacity to shift the focus from curative care to health promotion and disease prevention, and to thereby reduce pressure on health and care services.

Yet, for social prescribing models to reach their full impact potential, the quality of evidence must improve. Studies should develop and file clear design protocols specifying pathways to impact and outcomes to be measured before programme implementation begins, accounting for potential intervening and contextual factors, and striving to achieve measures for comparative control groups. Employing good practices at both the implementation and the evaluation stages will benefit participants in person-centred care systems as well as researchers who engage in the comparative study of public health.

## Conclusion

Our study broadens current literature in two key respects. First, we are one of the first reviews to utilise NHS England and Drinkwater et al.’s guidelines^8,13^ to examine the evidence of social prescribing impact on the individual, community, and health/care system. Second, we are the only review to our knowledge to assess the evidence of social prescribing specifically as it addresses the ‘loneliness epidemic’. Our findings show that individuals and organisations view social prescribing initatives as useful and necessary to tackle loneliness. However, given the wide variation in social prescribing interventions and how/whether their impact is investigated, it is difficult to draw definite conclusions regarding the effectiveness of these initiatives on individuals, communities, and health/care systems in general.

Similar to previous social prescribing research, our review highlights a fundamental need for consensus on what constitutes good impact evidence with respect to social prescribing.^8,14,16,22^ We demonstrate a gap between social prescribing design and social prescribing evaluation and illuminate a lack of impact assessment in relation to social care. We also note a lack of consensus on what the impact of a person-centred approach such as social prescribing should be. Social prescribing is presented as a person-centred, holistic, integrated approach to addressing individual needs, meaning impact on the whole person, including social service usage, should be studied.

Futhermore, we note a need for methodological and conceptual clarity in relation to loneliness and related concepts such as social isolation. Being able to distinguish between these related phenomena is an essential first step for mapping out needs and services required to help lonely individuals, who are likely to feel alone even in a crowd. Improved impact evidence is needed to know best how to reach lonely individuals and address complex health and social needs that emerge as a result of loneliness. In particular, we note the need to study links between an individual’s level of loneliness and one’s health and social care usage, as well as the impact of these individual attributes on one’s wider community.

We are compelled to point out that the COVID-19 pandemic has changed both the way person-centred care such as social prescribing is and can be delivered, and the ways in which such programmes fit into the larger health picture. In particular, much social prescribing in the UK is now being delivered through digital tablet, telephone, and email, with link workers connecting participants to social outlets virtually, helping to coordinate prescription delivery, and providing ways for people to connect to their communities while observing pandemic-related restrictions.^36^ Importantly, social prescribing has also reportedly eased much of the burden GPs expected to encounter during pandemic management, as GPs have been able to refer patients to social prescribing services based on telephone consultations, without causing anyone to physically attend a GP appointment.^37^ It thus appears that social prescribing is filling the role it was originally intended to have. Systematic and rigorous evaluations to this effect are long overdue.

## Limitations

Our review includes the most recently available evidence on social prescribing. All of the studies were conducted from 2014 to 2019 in the UK. Although our search was not limited geographically or to this date range, our findings suggest that the ‘social prescribing’ nomenclature is not utilised regularly outside the UK, Canada, and a few select places, and/or that social prescribing programmes are rarely assessed in terms of their impact on loneliness. Our work also demonstrates that the UK initiative to deliver person-centred care through social prescribing can only be based on evidence from the past 5 years.
